# Role of Omega 3 Fatty Acid as an Adjunct Treatment to Depression in Different Age Groups of the Patient Population - A Current Literature Review

**DOI:** 10.1192/j.eurpsy.2024.1129

**Published:** 2024-08-27

**Authors:** S. Poudel, A. Dhawan, V. Vijayakumar, S. Flores, A. Abozaid, K. Parajuli, R. Aggarwal, A. Sadana, H. Kumar, J. Choudhari, N. Panta, S. S. Ahmed

**Affiliations:** ^1^Department of Research & Academic Affairs, Larkin Community Hospital, South Miami, United States; ^2^Gian Sagar medical College, Patiala, India; ^3^American University of Barbados, St. Micheal, Barbados; ^4^University of Medicine & Health Sciences, St. Kitts & Nevis, American Samoa; ^5^Faculty of Medicine, Tanta University, Tanta, Egypt; ^6^Adesh Institute of Medical Science and Research, Bhatinda, India; ^7^Dow University of Health Sciences, Karachi, Pakistan; ^8^Kathmandu Medical College, Kathmandu, Nepal

## Abstract

**Introduction:**

Depression is a widespread problem that affects individuals of all ages. This study looks at the use of omega-3 polyunsaturated fatty acids (PUFAs) as an additional therapy for depression in people of different ages. Depression has an impact on everyone, from youth to the elderly, causing therapeutic concerns such as treatment resistance and recurrence. Omega-3 PUFAs, which may be found in fish and flaxseed, are important because of their impact on neurochemistry, inflammation, and neuroprotection. While pharmacotherapy, including antidepressants, has proven beneficial for many, the likelihood of remission and recurrence remains substantial. In recent years, there has been a growing interest in the potential role of omega-3 polyunsaturated fatty acids (n-3 PUFAs) in mitigating depressive symptoms. The primary constituents of n-3 PUFAs are eicosapentaenoic acid (EPA) and docosahexaenoic acid (DHA). Understanding the potential of omega-3 PUFAs across the lifespan can help address the multifaceted challenges posed by depression and improve mental health outcomes for diverse age groups.

**Objectives:**

This review aims to assess the role of omega-3 fatty acids in depression treatment across different age groups: children and adolescents, adults (18–60), and the elderly (60+). It investigates the effectiveness and potential differences in omega-3 supplementation among these age cohorts.

**Methods:**

A comprehensive literature search was conducted from 2003 to 2023 using PubMed, Google Scholar, and EMBASE, using specific keywords. Studies with inadequate age group information or Omega-3 intervention were excluded.

**Results:**

In children and adolescents, several studies indicate a positive association between omega-3 supplementation and improved depressive symptoms. In adults, results are mixed, with some studies showing benefits while others do not. In the elderly, omega-3 PUFAs appear to have a more consistent positive effect on depression. In contrast, a consistent positive association was observed in the geriatric population, suggesting that Omega-3 PUFAs may hold particular promise in the treatment of depression among older adults. However, variations in methodology, dosage, and study populations contribute to these mixed findings.

**Conclusions:**

Omega-3 PUFAs show promise as an adjunct therapy for depression across different age groups. Further research with standardized methodologies and larger sample sizes is needed to clarify their role and establish optimal dosage guidelines. Omega-3 PUFAs should be considered as a potential complement to conventional depression treatments, emphasizing the need for personalized approaches in depression management.

**Disclosure of Interest:**

None Declared

